# Perceptions and Practices in Parents of Saudi Children with Asthma: A Cross-Sectional Survey

**DOI:** 10.7759/cureus.2213

**Published:** 2018-02-21

**Authors:** Amani Abu-Shaheen, Isamme AlFayyad, Abdullah Nofal, Mohamad Al-Tannir, May AlMadaney, Humariya Heena

**Affiliations:** 1 Research Center, King Fahad Medical City, Riyadh, Saudi Arabia; 2 Disaster Management Unit, King Saud University Medical City

**Keywords:** asthma, dyspnea, emergency department, pediatrics, saudi arabia

## Abstract

Objective:

To acquire more precise data on perceptions and practices adopted by Saudi parents of asthmatic children regarding asthma and its management.

Methods:

A cross-sectional study was conducted through 2015 on 292 parents of children (aged 3–15 years) with asthma visiting the outpatient clinics and the emergency departments (ED) of two tertiary care medical centers in Riyadh city, using a self-administered questionnaire.

Results:

Out of 292 parents who participated in this study, 60.2% reported that their children had previously difficulty in sleeping at night due to an asthma attack. The majority (70.4%) of parents was worried about adverse effects of inhaled corticosteroids, and 58.8% of participants were worried about other inhaler adverse effects, whereas 29.0% believed that their child would develop a dependency on asthma medications. Around 82% reported visiting the pediatric emergency department for asthma treatment and 61.2% of participants reported going to the routine physician follow-up visits. Family income was significantly associated with parental concerns about the adverse effects of inhaled medications and corticosteroids as well as drug dependency (p = 0.044, p = 0.033, and p = 0.001, respectively). One hundred and seventy (57%) of the children used inhaled β-agonists while only 39 (13.3%) were using inhaled corticosteroids.

Conclusions:

Participated parents had misperceptions regarding the use of asthma medications and thus adopted ineffectual practices in its management. Therefore, to enhance asthma care and compliance in children, it is essential to develop different comprehensive parental education programs.

## Introduction

Asthma has been long recognized as a major public health problem, affecting a large chunk of the world population [1-4]. The urbanization and industrial development have led to an upsurge in the incidence of asthma, not only in adults but also in children who are adversely affected [1-4]. As Riyadh is the fifth-most (air) polluted city in the world, asthma is one of the most commonly diagnosed diseases in the pediatric emergency departments (ED) [5]. Most of the asthmatic children brought to the ED present with either recurrent or uncontrolled asthma symptoms. Poor control of childhood asthma is historically attributed especially in developing countries to a number of factors, including the ignorance of parents about the disease, the exposure of children to an adverse environment, a lack of compliance with medications, and infrequent visits to clinics for asthma control [6-10]. The most striking findings supporting this notion were from the survey titled "Asthma Insights and Reality in the Gulf and the Near East", which found that parents tended to underestimate the severity of their child’s asthma and overestimate the degree of asthma control [11-12]. However, a significant role is also played by many fears and concerns that the parents have regarding asthma medications and their side effects, including dependency. Parents’ attitudes and beliefs are important determining factors for appropriate medication use. Appropriate treatments for asthma not only rely on accurate evaluation and prompt intervention by the physician but also on proper home management among caregivers and parents [5] as serious lapses in asthma care by caregivers can have serious repercussions on morbidity and mortality. The successful management of childhood asthma requires a comprehensive approach and involves a ‘therapeutic partnership’ between the parents and the healthcare provider.

The steps needed to improve asthma control, in general, include raising the standard of clinical care and better patient adherence to prescribed medication, both of which require patient education and awareness programs. Implementation of these programs would increase the cost-effectiveness of asthma care in children and reduce the socio-economic burden of this disease on society.

We performed a study to understand the parental perception and practice for childhood asthma through a community-based study where children with wheeze and respiratory symptoms were categorized as asthmatic, and their parents were questioned about their perceptions regarding childhood asthma. The authors reported their findings in over 1450 respondent parents in various schools of Riyadh and were stratified according to the five districts of the city [13]. The present study was undertaken to acquire more precise data on perceptions and practices adopted by Saudi parents of asthmatic children regarding asthma and its management, as the parents participating in this hospital-based study had children with clinically diagnosed asthma confirmed by medical record review rather than only indeterminate respiratory symptoms.

## Materials and methods

Study design and setting

A cross-sectional study was conducted at two hospitals in Riyadh, Saudi Arabia through 2015; King Fahad Medical City (KFMC) and King Khalid University Hospital (KKUH). The participants were parents of children with asthma aged 3–15 years visiting the outpatient clinic and ED at KFMC or KKUH and were willing to participate in the study.

Recruitment of study participants

The predetermined prevalence of asthma in the Saudi pediatric population is 25% [7]. Therefore, the sample size was determined using a prevalence of 25% with 5% margin of error at 95% confidence interval (CI). The requisite sample size for the study was calculated to be 289. Investigators invited parents of children with asthma in the waiting rooms of the outpatient clinics and ED at KFMC and KKUH over a period of seven months, recruiting 292 participants. A trained research assistant enrolled voluntarily willing parents who filled in a questionnaire and returned it.

Questionnaire

A self-administered questionnaire was used to assess the perceptions and practices of parents toward asthma and its management in their children [8]. To validate the questionnaire, a pilot study was conducted with the first 30 participants, where Cronbach’s alpha was >0.7.

The final questionnaire consisted of three sections. The first section composed of the demographics (age, sex, area of residence, educational level, the number of children in the family, family income, and history of asthma), consequences of asthma attacks (school absenteeism, difficulty sleeping at night, hospitalization), and symptoms of uncontrolled asthma. The other two sections assessed the perceptions and practices of the parents towards asthma and its management. The questionnaire assessed the parents for the perceived etiology of asthma, its trigger factors, adverse effects of medications, and their fears about the child’s dependency on medication. The questions were in an open-ended multiple-choice format.

The ethical approval was obtained from the respective Institutional Review Boards at KFMC and KKUH and written informed consent was obtained from the participants.

Data analysis

Data were analyzed using SPSS version 21.0 (IBM Corporation, Armonk, NY, US). Descriptive statistics (mean, standard deviation, and percentages) were used to describe the quantitative and categorical variables. Pearson’s chi-squared test was used to compare the distribution of categorical variables. A p-value <0.05 was considered significant‏.

## Results

The demographic and disease history data for the 292 parents of asthmatic children are shown in Table 1. The mean age of the children parents was 36.66 ± 3.6 years. A family history of asthma was reported in 61.7% of the families.

**Table 1:** Sociodemographic characteristics of parents. One Saudi Riyal equals 0.27 US Dollar

Characteristics	n (%)
Sex	
Male	174 (59.6)
Female	118 (40.4)
Area of residency	
Urban	243 (83.5)
Rural	48 (16.5)
Parental education level	
Illiterate	19 (6.6)
Secondary	120 (41.5)
University	128 (44.3)
Higher Education	22 (7.6)
Monthly family income in Saudi Riyals	
<10,000 (Low)	135 (48.4)
10,000–19,999 (Middle)	100 (35.8)
≥20,000 (High)	44 (15.8)
Number of children in the family	
1–5	146 (51.6)
6–10	122 (43.1)
>10	15 (5.3)
Family history of asthma (n = 292 respondents)	179 (61.7)

One hundred seventy (57%) children used inhaled β-agonists while only 39 (13.3%) were using inhaled corticosteroids. The use of leukotriene antagonists for prophylaxis against acute asthma was used by only 6.5% of the participating asthmatic children (Table 2).

**Table 2:** Asthma medications use by the asthmatic children of the participating parents.

Medication	n(%)
Inhaled β-agonists	170 (57%)
Inhaled corticosteroids	39 (13.3%)
Leukotriene antagonists	19 (6.5%)
Antibiotics	3 (1.02%)
O_2_	3 (1.02%)
Antihistamine	2 (0.7%)
Oral Steroids	2 (0.7%)
Combined (Inhaled β-agonists & inhaled corticosteroids)	2 (0.7%)

When asked about the symptoms and consequences of uncontrolled asthma in children, the top two symptoms were nocturnal dyspnea (122, 42.2%) and repetitive cough (89, 31.8%).

One hundred and fifty-seven (60.2%) parents reported that their children had previous difficulty in sleeping at night due to an asthma attack (Table 3).

**Table 3:** Symptoms and consequences of uncontrolled asthma.

Symptoms of uncontrolled asthma	n (%)
Nocturnal dyspnea (more than one night/week)	122 (42.2)
Repetitive cough (daily)	89 (31.8)
Exercise dyspnea	86 (31.2)
Dyspnea at rest	70 (25.5)
Consequences of asthma	
Disturbed sleeping (once /month)	157 (60.2)
School absenteeism (once /month)	155 (59.6)
Hospitalization	168 (58.1)

The perceptions of participants about asthma are shown in Table 4. One hundred and forty-three (52.8%) participants believed that asthma has a hereditary etiology and dust (206, 70.8%) was the most commonly identified asthma-triggering factor. The majority i.e. 190 (70.4%) of the parents were worried about adverse effects of inhaled corticosteroids and 163 (58.8%) participants were worried from other inhaler adverse effects. Moreover, 80 (29.0%) believed that their child would develop a dependency on asthma medications.

**Table 4:** Perceptions about asthma among parents of children with asthma.

Parental beliefs	n (%)
Etiology	
Hereditary	143 (52.8)
Acquired	21 (9.2)
Other	74 (33.5)
Triggers	
Dust	206 (70.8)
Indoor smoking	83 (28.4)
Virus	120 (41.1)
Food	43 (14.8)
Worries about medications	
Inhaled corticosteroids adverse effects	190 (70.4)
Other inhaler adverse effects	163 (58.8)
Dependency	80 (29)

Around two-thirds of parents responded that they were able to manage their child’s asthma at home, whereas 82.1% reported visiting the pediatric emergency department for asthma treatment in the past one year, and 61.2% of participants going to the routine physician follow-up visits (Table 5).

**Table 5:** Asthma practices among parents of children with asthma * A medicinal substance converted into vapor on boiling with water and inhaled to open the airways.

Parental practices	n (%)
Able to treat asthma at home	188 (67.6)
Treatment used	
Vapor^*^	173 (64.1)
Asthma Medications	115 (42.6)
Massage	34 (12.6)
Herbs	38 (14.1)
Certain foods	5 (1.9)
Precautions used	
Avoiding strong odor	230 (83.6)
Avoiding indoor smoking	235 (87.4)
Regularly using medicines	218 (80.1)
Avoiding cold weather	237 (87.1)
Avoiding physical exercise	162 (61.1)
Avoiding certain foods	90 (33.7)
Visiting pediatric emergency department in the past year	230 (82.1)
Routine physician follow-up visits	177 (61.2)

Table 6 shows the association between family income with the parental concern regarding the adverse effects of inhalers in general, and steroid use in particular, and dependency on drugs (p = 0.044, p = 0.033, and p = 0.001, respectively) which was statistically significant. No statistically significant associations were found between parental practices and the other demographic variables studied.

**Table 6:** Association between family income and concerns about the use of inhaled therapy in the parents of children with asthma.

Concerns	Family income (Saudi Riyals per month)			p-value
	<10,000 (Low) n (%)	10,000–19,999 (Middle) n (%)	>20,000 (High) n (%)	
Adverse effects of inhalers	86 (54.8)	52 (33.1)	19 (12.1)	0.044
Adverse effects of inhaled corticosteroids	99 (53.8)	62 (33.7)	23 (12.5)	0.033
Dependency	49 (66.2)	20 (27.0)	5 (12.2)	0.001

## Discussion

This study was necessitated by the fact that more precise data needs to be acquired to gauge the perceptions and practices adopted by Saudi parents in order to manage asthma in their children.

Parents and caregivers play a pivotal role in the management of their child’s asthma. Healthcare providers cannot force asthma treatment upon the children; it is the parents who decide whether they will follow medical advice or not. This parental decision is largely based on their perceptions of the illness and medication. In our study, we found that one hundred and seventy (57%) children used inhaled β-agonists while only 39 (13.3%) used inhaled corticosteroids. The use of leukotriene antagonists for prophylaxis against acute asthma was used by only 6.5% of the participating asthmatic children. These indicate participants' concerns about their child’s dependency and adverse effects of asthma medications in general, the effects of the long-term use of steroids in specific which might be related to poor knowledge regarding asthma, the absence of partnership in the management process, inappropriate anticipations, and cultural variances. It is well recognized that cultivating adherence to inhaled corticosteroids in children with asthma is perhaps the most effective way through which healthcare providers can lessen uncontrolled asthma burden [14]. However, the majority of parents with asthmatic children from Asian countries view corticosteroids inhaler as addictive or a dependency-causing drug, a misconception fuelled by extensive media campaigns about modern-day inhalational drug abuse and steroid abuse [15]. These concerns may likely prompt the children to miss the prescribed doses of inhaled corticosteroids with increased problems of non-adherence and frequent hospitalizations. Parents from western countries mostly base their childrens' inhaled corticosteroids maintenance remedies of the children according to their child’s health need, the anticipated level of harm from medications, and the convenience of medications administration in contrast to the practices adopted by their counterparts in developing countries [13]. A previous study of 170 parents of children with asthma showed that 66% had concerns relating to inhaled therapy, including the adverse effects of asthma medications (91%), and inhaler dependency [16]. Qualitative studies’ findings have shown how parents take the medical care of their child’s asthma into their own hands by balancing the perceived need for inhaled corticosteroids against their concerns about (side effects) of medication [17-19]. This finding emphasizes the importance of parental perceptions about illness and medication. Ignorance among parents about the underlying nature of asthma and unrealistic fears about side effects from regular anti-inflammatory controller medication constitute significant barriers. Improved understandings about the perceived side effects of medications in parents are considered as fundamental to asthma care. Parental education through either community-based campaigns or health-care professionals could positively change their beliefs about medication use and asthma management in their children.

Regarding parental practices in asthma control, parents usually avoided strong odors or used vapor treatment and only a few used appropriate asthma medications. This stresses the importance of a strong doctor–parent partnership where such practices could be discussed and modified. Another important observation was that higher number of parents visited pediatric emergency department on their child’s asthma attack rather than attend the routine follow-up clinic. This leads to increased pediatric emergency hospitalization and calls for improper health care utilization causing undue burden. Consistent with other studies [10-11, 20], our results also show that parents reported a high rate of disturbed sleep nocturnal dyspnea, repetitive cough, school absence, and hospitalization due to asthma. These findings imply that asthma control and management in our settings should aim at imparting awareness among parents using guidelines.

 Parents with lower family incomes were more concerned more about medication dependency and the side effects of inhaled corticosteroids, which constitute a major barrier to asthma care and adherence to treatment among low-income families and caregivers [20-21]. 

We suggest that health care providers and educators modify such perceptions by offering regular asthma awareness as per guidelines through one to one consultations in the clinics, distributing pertinent pamphlets, brochures to the parents, and conducting educational campaigns. This approach may help to improve adherence and increase asthma control in the children.

The strengths of this study was that the diagnosis and medication here were confirmed by physician diagnosis and patient medical records, unlike our previous epidemiological study [13]. Moreover, the current study was conducted in two major tertiary hospitals in Riyadh city KFMC and KKUH. These hospitals provide exemplary care and a commitment to overall quality of life for a wide spectrum of Saudi population across the country.

Study limitations: In cross-sectional studies, it is difficult to make causal inference. Besides, participants’ recall bias and responses exaggeration cannot be excluded. The generalizability of the study findings may not be applicable to the rest of Saudi Arabia. Selection bias is a significant limitation that should be considered in future studies, as a large percentage of parents reported visiting ED in the past one year, reflecting the severity of the disease in their children. It is likely that parents of children with severe asthma have more misconceptions, malpractices, and less compliance than parents whose children do not visit the ED.

## Conclusions

Participated parents had misperceptions regarding the use of asthma medications and adopted ineffectual practices in its management. Therefore, to enhance asthma care and compliance in children, it is essential to develop different comprehensive parental education programs.
